# Infectious Aortitis: Case Report and Literature Review

**DOI:** 10.7759/cureus.23198

**Published:** 2022-03-15

**Authors:** Basel Abdelazeem, Soumya Kambalapalli, Abdelilah Lahmar, Amman Yousaf, Halina Kusz

**Affiliations:** 1 Internal Medicine, McLaren Health Care, Michigan State University (MSU), Flint, USA; 2 Medicine, Faculty of Medicine and Pharmacy of Oujda, Oujda, MAR; 3 Internal Medicine/Geriatrics, McLaren Health Care, Michigan State University (MSU), Flint, USA

**Keywords:** literature review, case report, infection, aneurysm, aortitis

## Abstract

Aortitis is the inflammation of the aorta secondary to either infectious or non-infectious etiologies. Infectious aortitis is a rare but potentially life-threatening condition. It is more common among older patients with preexisting pathology. Clinical presentation is variable, therefore, a high index of suspicion is required for timely diagnosis and management. We report a case of aortitis which was complicated with the development of a saccular abdominal aortic aneurysm. A 76-year-old male presented to the Emergency Department with two days of right lower quadrant abdominal pain. Clinical evaluation and imaging studies revealed abdominal aortitis, which progressed to a saccular abdominal aortic aneurysm. We highlight a unique presentation of infectious aortitis to raise awareness among physicians. We also reviewed the available literature on infectious aortitis to illustrate the importance of early diagnosis and appropriate treatment to improve the patients' outcomes.

## Introduction

Aortitis is the inflammation of the aortic wall, which may be localized to isolated parts of the aorta, such as thoracic or abdominal, or may involve the entire length of the aorta. It is reported as a rare disorder, but the actual incidence may be underestimated [1]. Aortitis may present with nonspecific constitutional symptoms, such as fever, loss of appetite, weight loss, or localized abdominal or back pain. The symptoms can occur over a long time period which can sometimes delay the patients from seeking medical advice until aneurysmal development and its rupture, which is often diagnosed at this stage. Patients with a ruptured abdominal aortic aneurysm (AAA) may present with hemorrhagic shock or septic shock due to infection/sepsis, manifesting as tachycardia and hypotension. This can complicate cardiovascular collapse [2,3].

We presented a case of an elderly patient admitted to the hospital because of abdominal pain. Initially, the imaging showed a malignant-looking mass in the cecum with severe adjacent inflammatory changes extending to the proximal aorta without aneurysm formation. The patient was diagnosed with abdominal aortitis with the formation of a saccular aneurysm formation within a couple of days. Few cases of aortitis with aneurysmal formation with variable patient presentations have been reported in the literature [4-10]. We aim to highlight the different etiologies, diagnostic tools, and management approaches for aortitis to emphasize the importance of early detection and proper management of aortitis.

## Case presentation

A 76-year-old Caucasian man presented to the emergency department with a chief complaint of sharp abdominal pain for the last two days located in the right lower quadrant associated with high-grade fever, chills, rigors, constipation, and unintended weight loss of six pounds in the previous month. The patient otherwise did not endorse any significant past medical history. The family history was pertinent of colon cancer in his mother. The social history was notable for 40-pack-years, however, the patient quit 20 years ago.

**Figure 1:**
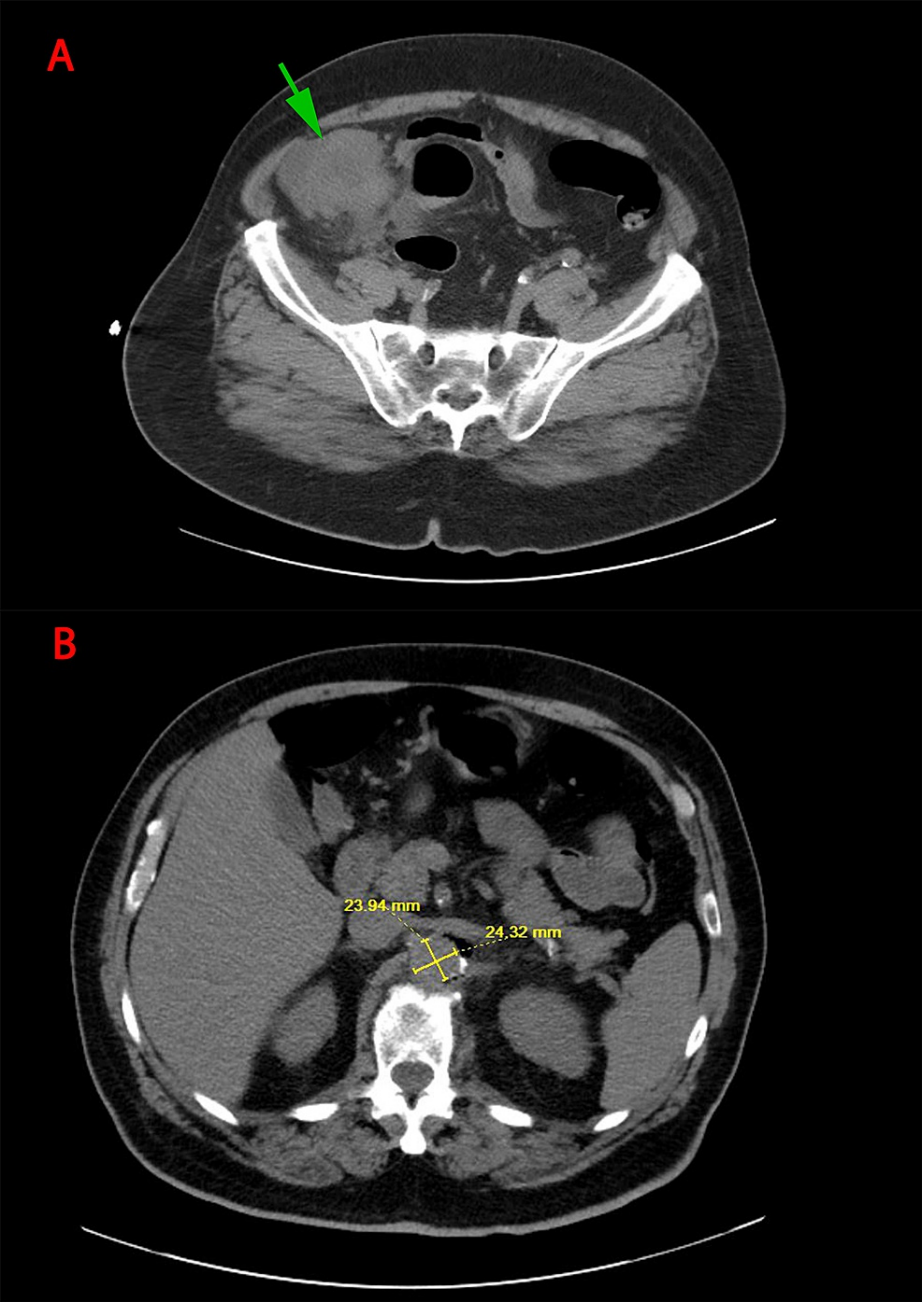
Computed tomography scan of the abdomen and pelvic at admission. A:  Mass in the cecum (green arrow) extending to the appendix and the ileocecal junction with severe adjacent inflammatory changes B:  Acute inflammatory changes were observed in the proximal abdominal aorta

**Figure 2:**
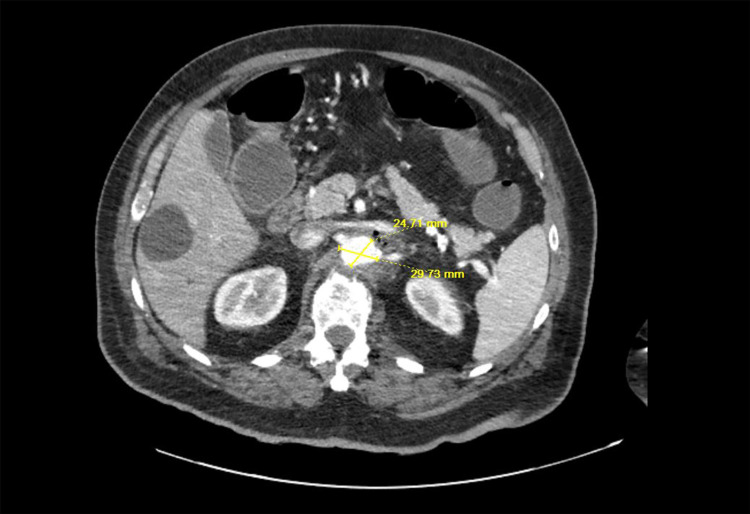
Computed tomography angiography of the abdomen showed mid abdominal aortitis

Vitals sign at presentation revealed a blood pressure of 176/113 mmHg, heart rate of 119 bpm, a body temperature of 100.3 ℉, and oxygen saturation of 96% under ambient oxygen conditions. The patient was conscious and alert. Physical examination was relevant for right lower quarter tenderness without hepatosplenomegaly. The laboratory findings are summarized in Table 1. The computed tomography (CT) scan of the abdomen and pelvis showed a mass in the cecum extending to the appendix and the ileocecal junction with severe adjacent inflammatory changes with no clear evidence of the fistula (Figure 1A). Acute inflammatory changes were observed in the proximal abdominal aorta (at the level of the superior mesenteric artery, left renal artery) with adjacent soft tissue air and air within the aortic wall, which was suspicious as an etiology of acute aortitis (Figure 1B). CT angiography was performed and revealed that the cecal mass was suspicious for malignancy. The patient was then recommended a colonoscopy for the suspicious cecal mass. In addition to several non-specific hepatic hypodensities, mid abdominal aortitis with a thickness of 2 centimeters was noted (Figure 2).

**Table 1:** Laboratory workup H: high; L: low; WBC: white blood cells

Lab	Value	Reference Range
WBC Count	15.9 H	4.50-11.00 X 10*3/uL
Absolute Neutrophils	13.0 H	1.40-6.50 X 10*3/uL
Hemoglobin	12.7 L	13.5-17.7 g/dL
Platelet Count	263	140-440 X 10*3/uL
Creatinine	1.09	0.50-1.50 mg/dL
Carcinoembryonic antigen	<0.5	0.0-4.9 ng/mL
Carbohydrate 19-9	<1.2	0.0-34.9 U/mL
Procalcitonin	0.16 H	0.02-0.09 ng/mL

The patient was admitted and treated with broad-spectrum antibiotics (piperacillin/tazobactam and vancomycin) and intravenous fluids as a treatment for sepsis. General surgery, vascular surgery, and the gastrointestinal specialty team were consulted. Subsequently, the patient underwent a colonoscopy, which indicated a large friable, malignant-looking cecal mass extending into the base of the terminal ileum valve. Then the patient underwent a diagnostic laparoscopy along with adhesion lysis and lymph node biopsy. The plan was made for a tentative right hemicolectomy. The patient was diagnosed with a moderately differentiated metastatic adenocarcinoma of the cecum through several biopsies.

**Figure 3:**
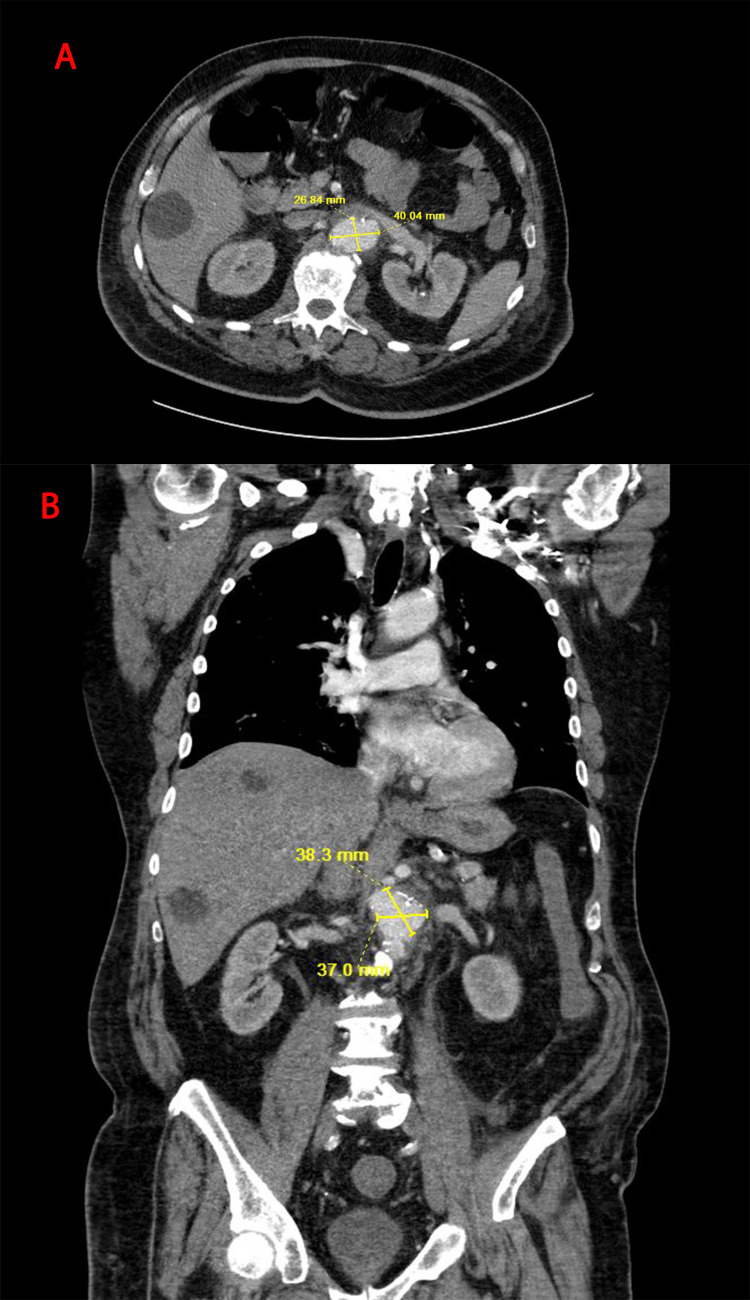
Computed tomography scan of the abdomen and pelvic Computed tomography scan of the abdomen and pelvic at hospital day six showed a new around 4 x 3 cm saccular aortic aneurysm from the left aspect of the proximal abdominal aorta. A: Axial section. B: Coronal section

**Figure 4:**
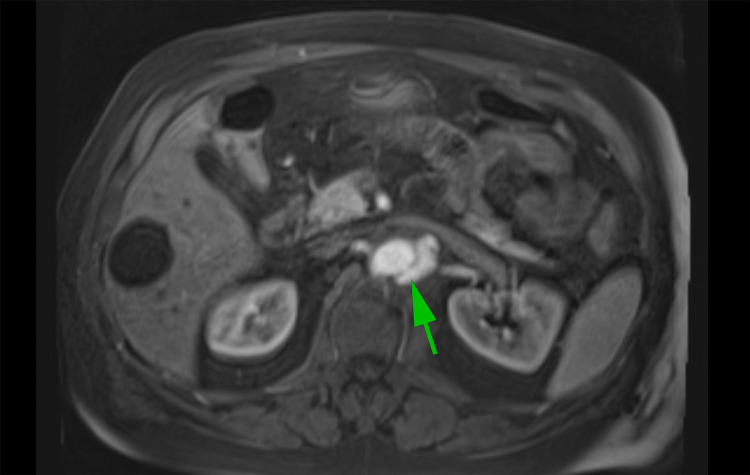
Magnetic resonance imaging revealed a mycotic abdominal aortic aneurysm

Overnight, the patient started having high-grade fever with a temperature maximum of 102 F, despite being on broad-spectrum antibiotics, with the patient reporting persistent abdominal pain. A repeat abdominal CT scan showed a saccular aortic aneurysm measuring 4 x 3 cm from the left aspect of the proximal abdominal aorta, which was new compared to the previous CT scan (Figure 3). Overall, the aorta measured 4 x 3 cm in axial dimension at the level compared to 2.5 x 2.3 cm in the previous CT scan. Magnetic resonance imaging (MRI) revealed a mycotic abdominal aortic aneurysm, and the overall aorta measures 4.0 x 2.9 cm in axial dimension (Figure 4). Blood cultures were negative for bacteria or fungi and the patient was started empirically on anidulafungin in addition to broad-spectrum antibiotics. Surgical intervention was discussed with the patient and the family. But in the meantime, the patient was diagnosed with acute hypoxic respiratory failure as a result of COVID-19 pneumonia which required average volume-assured pressure support (AVAPS). The patient was transferred to the intensive care unit for closer monitoring.

The patient also developed thrombocytopenia likely secondary to COVID-19 as the heparin-induced thrombocytopenia panel was negative. The patient was given intravenous immunoglobulin therapy, steroids, and multiple platelet transfusions, as well as being closely monitored for any signs of bleeding.

The patient developed a multi-organ dysfunction, with acute kidney damage, gastrointestinal bleeding with burgundy-colored stool, worsened respiratory status despite a fraction of inspired oxygen (FiO2) of 100% on AVAPS, rapid desaturation even with minimal movement, worsening of serial chest x-rays with diffuse bilateral ground-glass opacities, consolidation zones, and air space opacities. The patient became hypotensive, and pressors were started to treat the septic shock. The patient's clinical condition deteriorated rapidly, so the multidisciplinary team discussed the clinical situation, the prognosis, and the different treatment options with the patient and his family. The decision was made to change his code status to comfort measure only. The patient expired shortly afterward. Table 2 summarizes the patient's timeline.

**Table 2:** Timeline

Time	Events
One month before the presentation	The patient had an unintentional weight loss of six pounds in one month.
Two days before admission	The patient started experiencing right lower quadrant abdominal pain, high-grade fevers with chills and rigors, and constipation.
Hospital day 1	The patient was admitted to the hospital. The computed tomography (CT) scan of the abdomen and pelvis showed a mass in the cecum extending to the appendix and the ileocecal junction with acute inflammatory changes in the proximal abdominal aorta.
Hospital day 2	CT Angiogram was performed and revealed that the cecal mass was suspicious of malignancy. Mid abdominal aortitis with a thickness of 2 centimeters
Hospital day 3	Colonoscopy was performed, revealing a large friable malignant-looking mass seen in the cecum extending into the base of the terminal ileum valve.
Hospital day 5	The patient underwent diagnostic Laparoscopy with lysis of adhesions and a lymph node biopsy.
Hospital day 6	The patient developed a high-grade fever overnight and persistent abdominal pain. CT scan revealed a new saccular aortic aneurysm and a prior detected cecal mass.
Hospital day 7	MRI revealed a mycotic abdominal aortic aneurysm demonstrating an increase in size by 1 mm than the previous scan.
Hospital day 8-19	The patient developed acute hypoxic respiratory failure as a result of COVID-19 pneumonia and multi-organ dysfunction. A discussion was made with the patient and his family regarding the prognosis and the different treatment options. Finally, the decision was made to go for comfort care.
Hospital day 20	Patient expired.

## Discussion

Aortitis can be caused by both infectious and non-infectious etiologies. Bacteria such as Streptococcus pyogenes, Streptococcus pneumonia, Staphylococcus, and Salmonella species are the most prevalent infectious causes. In contrast, giant cell arteritis, Takayasu arteritis, and different rheumatological diseases are the most common non-infectious causes [11]. The aorta, as a major blood vessel, is more resistant to damage or destruction compared to other body blood vessels. However, a few factors can weaken the aortic wall, cause aneurysms, and also predispose the aorta to infectious etiologies. Those factors include uncontrolled diabetes mellitus or hypertension, cancer, iatrogenic inoculation as a result of surgical intervention or the use of medical equipment or devices in and around the aorta, atherosclerotic diseases, vascular malformations, or medial cystic necrosis of the vascular wall. The route by which aortitis occurs includes hematogenous seeding of an existing intimal injury, septic emboli, direct spread from an infectious site, and bacterial inoculation. Still, it can be challenging to identify a primary source of infection, and a definitive conclusion is only reached in half of the reported cases [12].

Infected aortic aneurysms, also known as mycotic aortic aneurysms (MAA), represent 0.7% to 26% of all aortic aneurysms [13]. MAA is most commonly caused by hematogenous bacterial growth in an existing aneurysm or an adjacent site of infection. After the colonization of the altered vascular endothelium, the collagenolytic and elastolytic enzymes are activated, and this stimulates suppuration and ultimately the formation of aneurysms, which can occur within a few weeks. COVID-19 is known to trigger activation of metalloproteinases and digestion of the collagen of the aneurysmal wall which can sometimes aid in the development of aneurysms [14].

There are no precise laboratory tests that can be utilized to determine the diagnosis of MAA. Blood cultures are vital clues, but they aren't always definitive, and they're only positive 50% to 82% of the time [15]. CT angiography is currently the best imaging technique for diagnosing mycotic aneurysms [13,16-18]. This enables an early diagnosis with the help of a high-resolution three-dimensional representation of the vascular anatomy for surgical or endovascular treatment planning and helps to recognize impending complications. Large aneurysms are plainly visible on contrast-enhanced CT, but small lesions can be missed. CT imaging manifestations of infectious aortitis include loss of aortic wall contour and thickening of the aortic wall due to surrounding inflammatory infiltrate, periaortic fluid accumulation, new or rapidly growing saccular aneurysm, or pseudoaneurysm, and occasionally air in the aortic wall [19]. In addition, CTA and MRA are also used as accurate imaging modalities. In particular, the CTA enables the early detection of changes in the vascular wall and thus a faster diagnosis [1,19].

Delayed diagnosis or non-treatment of mycotic aneurysms often leads to high morbidity and mortality from sepsis or life-threatening bleeding [13]. It should also be noted that the patient's infection with COVID-19 had additionally worsened the patient's prognosis because of the multiorgan failure that the affected patient may have. This is what has been reported by various studies which have objectified a high rate of rupture of abdominal aortic aneurysms which can delay treatment and increase mortality in some patients. In addition, it is always necessary to rule out the other etiologies of aortitis, in particular of non-infectious origin, before retaining the final diagnosis. When infected aortitis is suspected, broad-spectrum antibiotics and vascular surgery consultation should be sought as soon as possible. Broad-spectrum antibiotics are started after blood cultures and prior surgery, ideally, as soon as the diagnosis is suspected and modified as needed depending on blood culture findings to reduce inflammation and improve local surgical conditions.

A multidisciplinary approach is recommended for the treatment of MAA, which must be individually tailored for each patient based on the characteristics of the aneurysm, such as its location, morphology, and associated complications. Different treatment options include open surgery, endovascular procedures (stent versus embolization), medical treatment, or a combination of these options [13,20]. The surgical approach should be adapted depending on the degree of contamination; with broad debridement and the collection of suitable intraoperative cultures, it includes an extra anatomical bypass graft or in situ graft. In hemodynamically unstable patients with aortic rupture, endovascular aneurysm repair might be used as a temporary treatment for patients with high risks for open surgical repair [2].

On the other hand, small asymptomatic aneurysms with no signs of extension or malperfusion are usually managed conservatively with long-term parenteral antibiotic therapy. But, according to a retrospective study, around 75% of conservatively treated patients died in the first three years after diagnosis [12]. There is no recommended duration of antibiotic therapy for mycotic aneurysms. It usually depends on the causative microorganism, the characteristics of the aneurysm, its location and size, and the patient's immunological status [20].

Mycotic aneurysms have a very poor prognosis, leading to increased mortality due to an impending rupture with life-threatening bleeding or complications due to the infection or the aneurysm itself. In our patient, the vascular reconstruction surgery was extremely high risk as he developed acute hypoxic respiratory failure due to COVID-19 pneumonia leading to a multi-organ dysfunction. Therefore, after discussion with the patient and family, the decision was made to change the patient code status to comfort care.

Bacterial aortitis with mycotic aneurysm formation of the aorta has been reported in the literature. In recent years additional few cases with infectious aortitis have been reported. Table 3 summarizes the patients' presentation, imaging findings, and prognosis of those cases. In addition to the cases mentioned above, our case aims to raise awareness among physicians about aortitis and the importance of considering it as a differential diagnosis in patients presenting with abdominal pain.

**Table 3:** The patients' presentation, imaging findings, causative organism, and prognosis of the cases reported in the literature CT: computerized tomography; AAA: abdominal aortic aneurysm; MA: mycotic aneurysm; NR: not reported; POD: postoperative day

Author, year	Presentation	CT scan finding/ Organism detected	Management/ Prognosis
Torsteinsen et al. 2022 [4]	A 58-year-old European male presented with abdominal and back pain for eight weeks.	An 85 mm inflammatory AAA/ Borrelia afzelii	Open aortic repair/ discharged after one week.
Yan et al. 2021 [10]	A 70-year-old woman presented with one-week history of non-specific lower abdominal pain bilaterally.	Non-specific fat stranding around a mildly dilated aorta maximally measuring 3.0x2.7 cm/ Group B Streptococcus	In situ repair/ discharged on POD ten.
Wan et al. 2021 [9]	A 75-year-old man presented with pain in his right lower extremity.	Unruptured AAA / Peptoniphilus harei	Endovascular stent-graft implantation/ discharged on day 12 of admission
Hau et al. 2021 [6]	A 76-year-old man presented with fever and delirium	Aortic arch aneurysm of 4.7 cm/ Serogroup D Salmonella enteritidis	Endovascular graft repair/ discharged on POD 13
Gunawardena et al. 2021 [5]	A 67-year-old man presented with sudden onset, severe, left-sided lower abdominal pain.	Infrarenal AAA of 4.2cm/ Candida albicans	In situ repair of the aneurysm/ passed away on POD 13.
Kesiena et al. 2021 [7]	A 36-year-old female presented to the emergency room for evaluation of worsening right-sided lower back pain of 10 days duration.	A 2.5 cm contained ruptured mycotic abdominal aneurysm/ NR	Open surgical repair/ discharged home.
Tong et al. 2021 [8]	A 55-year-old female presented with abdominal discomfort and fever for a week.	Ruptured MA at the left external iliac artery and a leaking MA at the juxta-renal aorta / Burkholderia pseudomallei	Endovascular aortic repair/ Passed away after two months secondary to relapsed melioidosis with dissemination (bacteremia, gallbladder empyema, and pneumonia)
Tong et al. 2021 [8]	A 57-year-old man presented with left abdominal pain and constitutional symptoms for two months.	Multiple MAs involving the thoracic and abdominal aorta. / Burkholderia pseudomallei	Endovascular repair of the abdominal and thoracic aorta/ discharged.
Tong et al. 2021 [8]	A 57-year-old man presented with fever and constitutional symptoms for two weeks.	A depicted a widened mediastinum and a lobulated saccular MA arising from the aortic arch / Burkholderia pseudomallei	Endovascular aortic repair/ discharged

## Conclusions

Aortitis can present with non-specific clinical symptoms and can be a life-threatening condition. Aortitis requires immediate diagnostic workup, including labs, cultures, and most importantly, CT imaging to diagnose, locate, and assess the characteristics of the aneurysms and surveillance of treatment efficacy. Once infectious aortitis is diagnosed early antibiotic therapy and surgery are crucial. Despite early diagnosis and treatment, the mortality rate remains high. We presented a patient who was promptly diagnosed with abdominal aortitis which was complicated with aneurysm formation. The patient expired due to multiorgan failure. Interdisciplinary teamwork plays a significant role in early detection and the individualization of treatment modalities, and thus insight into the patient's prognosis.
